# Litchi Pericarp Extract Treats Type 2 Diabetes Mellitus by Regulating Oxidative Stress, Inflammatory Response, and Energy Metabolism

**DOI:** 10.3390/antiox13040495

**Published:** 2024-04-21

**Authors:** Ziming Yang, Li Zhang, Jinlei Liu, Dianpeng Li

**Affiliations:** 1 Guangxi Key Laboratory of Plant Functional Phytochemicals and Sustainable Utilization, Guangxi Institute of Botany, Guangxi Zhuang Autonomous Region and Chinese Academy of Sciences, Guilin 541006, China; zl@gxib.cn (L.Z.); ljl@gxib.cn (J.L.); 2 Engineering Research Center of Innovative Traditional Chinese, Zhuang and Yao Materia Medica, Ministry of Education, Guangxi University of Chinese Medicine, Nanning 530200, China

**Keywords:** type 2 diabetes mellitus, litchi pericarp, oxidative stress, inflammatory response, AMPK, Nrf2, NF-κB

## Abstract

Litchi pericarp is rich in polyphenols, and demonstrates significant biological activity. This study assessed the therapeutic effects of litchi pericarp extract (LPE) on type 2 diabetes mellitus in db/db mice. The results showed that LPE ameliorated symptoms of glucose metabolism disorder, oxidative stress, inflammatory response, and insulin resistance in db/db mice. The mechanistic studies indicated that LPE activates adenosine 5‘-monophosphate (AMP)-activated protein kinase (AMPK) and suppresses the protein expression of phosphoenolpyruvate carboxykinase (PEPCK), thereby reducing hepatic gluconeogenesis. Additionally, LPE facilitates the translocation of nuclear factor erythroid2-related factor 2 (Nrf2) into the cell nucleus, initiating the transcription of antioxidant factors superoxide dismutase (SOD) and NAD(P)H: quinone oxidoreductase 1 (NQO1), which alleviate oxidative stress and reduce oxidative damage. Furthermore, LPE blocks nuclear factor kappa-B (NF-κB) nuclear translocation and subsequent inflammatory response initiation, thereby reducing inflammation. These findings indicate that LPE addresses type 2 diabetes mellitus by activating the AMPK energy metabolic pathway and regulating the Nrf2 oxidative stress and NF-κB inflammatory signaling pathways.

## 1. Introduction

Type 2 diabetes is a metabolic disorder characterized by hyperglycemia. This condition is often linked with obesity, diabetic nephropathy, and cardiovascular diseases, posing significant health threats. In 2021, it was estimated that 537 million adults worldwide suffered from diabetes, equating to approximately 1 in 11 adults, with type 2 diabetes comprising around 90% of these cases [1]. Projections suggest a rise to 693 million by 2045, if current trends persist [2]. The origins of type 2 diabetes are complex, encompassing metabolic irregularities, poor dietary and lifestyle choices, and genetic factors [3,4]. Diet-related risks include excessive consumption and a high intake of fried foods and sweets. Lifestyle factors such as lack of physical activity, insufficient sleep, occupational stress, and adverse emotional states further elevate the risk of type 2 diabetes. Moreover, genetic predisposition is significant; individuals with a family history of diabetes are at an increased risk compared to the wider population [2].

The pathogenesis of type 2 diabetes mellitus remains unclear, with no single theory fully accounting for all instances. An imbalance in energy metabolism plays a pivotal role in its onset. Consequently, optimizing energy metabolism and minimizing energy accumulation are essential in managing the disease’s progression. Among the enzymes involved in this process, adenosine 5′-monophosphate (AMP)-activated protein kinase (AMPK) is of particular importance [5]. AMPK activation has been demonstrated to mitigate hepatic gluconeogenesis anomalies and stabilize glucose levels [6]. Oxidative stress, a physiological phenomenon where harmful substances like free radicals exceed the antioxidant capacity, leads to stress reactions and is a significant contributor to type 2 diabetes [7]. These radicals can inflict oxidative damage on biological macromolecules, potentially leading to disease manifestation or expedited aging [8,9]. Oxidative stress-induced type 2 diabetes primarily affects the insulin signaling pathway, particularly through lipid oxidative damage to the cell membrane where the insulin receptor resides, reducing insulin function and causing insulin resistance [10,11]. Studies indicate that oxidative stress markers, such as malondialdehyde (MDA), are markedly elevated in type 2 diabetes patients [12]. Thus, controlling oxidative stress is crucial in managing type 2 diabetes. The nuclear factor erythroid2-related factor 2 (Nrf2) signaling pathway is a key player in the antioxidative stress response [13]. Activation of the Nrf2 pathway encourages the expression of antioxidant-related genes. Consequently, Nrf2 pathway activation is being explored as a potential treatment for type 2 diabetes. Natural compounds that act as Nrf2 agonists have been effective in ameliorating type 2 diabetes mellitus. For example, grape seed proanthocyanidins have shown to bolster antioxidant defenses by activating the Nrf2 pathway, leading to decreased blood sugar levels and diminished renal damage in diabetic rats [14]. Similarly, okra polysaccharides have been found to enhance the expression of superoxide dismutase (SOD) and heme oxygenase 1 (HO1) through Nrf2 upregulation, thus exerting a therapeutic effect on type 2 diabetes [15]. Type 2 diabetes is also recognized as a mild, chronic, and systemic inflammatory disease [16]. Inflammatory factors are known to disrupt the insulin signaling pathway and foster insulin resistance [17]. Nuclear factor kappa-B (NF-κB), a pivotal element in the inflammatory response, plays a crucial role [18]. The activation of NF-κB leads to the transcription of pro-inflammatory cytokines, such as tumor necrosis factor-α (TNF-α), interleukin-6 (IL-6), and interleukin-1β (IL-1β), which in turn can further activate NF-κB, intensifying the inflammatory response and hastening the progression of diabetes.

The incidence of type 2 diabetes mellitus is anticipated to escalate significantly due to unhealthy lifestyle and dietary habits. This surge poses a considerable challenge to society, families, and individuals alike. Thus, it is critical to explore effective methods for the prevention and control of type 2 diabetes mellitus. Natural products have attracted significant interest in the medical realm. Notably, China, as the origin and leading producer of lychee, accounts for more than half of the global supply [19]. However, the processing of lychee results in the wastage of large quantities of skin, posing a challenge to the industry’s development. Studies have shown that the litchi pericarp is abundant in polyphenolic compounds, such as procyanidin A2, procyanidin B2, (−)-epicatechin, (+)-catechin, quercetin, (−)-epigallocatechin gallate, and rutin, with (−)-epicatechin being predominant [20,21,22]. The administration of powdered fresh lychee peels to diabetic rats has demonstrated a hypoglycemic effect [23]. Furthermore, litchi pericarp extract (LPE) from various lychee varieties has been found to enhance glucose consumption in HepG2 cells, with its effectiveness closely correlated with the (−)-epicatechin content [24]. Although the hypoglycemic properties of litchi peel have been reported, the mechanisms and active components involved remain unclarified. Previous studies have highlighted LPE’s potent antioxidant and anti-inflammatory properties [19,25,26,27], and this study aims to investigate the potential mechanisms by which LPE exerts hypoglycemic effects through the mitigation of oxidative stress and inflammatory responses.

## 2. Materials and Methods

### 2.1. Materials and Chemicals

Antibodies targeting glyceraldehyde-3-phosphate dehydrogenase (GAPDH), lamin B1, adenosine 5′-monophosphate (AMP)-activated protein kinase α (AMPKα), phospho-AMPKα (p-AMPKα), superoxide dismutase 1 (SOD1), Nrf2, NF-κB p65, NAD(P)H: quinone oxidoreductase 1 (NQO1), and phosphoenolpyruvate carboxykinase (PEPCK) were purchased from Cell Signaling Technology (Beverly, MA, USA). Feizixiao litchi was collected from an agricultural ecological park in Lingshan County, Guangxi. Male db/dm wild-type mice and male db/db type II diabetic mice, both 6 weeks old and SPF grade, were sourced from the Model Animal Research Center of Nanjing University (Nanjing, China), under certification number SCXK (Su) 2020-0001. Mouse feed was provided by Hunan SJA Laboratory Animal Co., Ltd. (Changsha, China), with a license under SCXK (Hunan) 2020-0002.

### 2.2. Preparation of LPE

Fresh lychee pericarp was soaked in an 80% ethanol solution at room temperature for 7 days, and then filtered. The filtrate was centrifuged at 3500 rpm for 10 min using a TGL-16R centrifuge (Heima, Zhuhai, China). The supernatant underwent column chromatographic purification through XDA-7 macroporous resin (conditions: the pH of the solution was 4.5 and the flow rate of the eluent was 6 BV/h), followed by elution with a 60% ethanol aqueous solution. Then, the eluate solution was concentrated under reduced pressure at 95 kPa and a heating temperature of 40 °C using a rotary evaporator (N-3100, Eyela, Tokyo, Japan). Subsequently, the concentrate was dried using an HF-015 spray dryer (Hefan, Shanghai, China) to yield the LPE. The HPLC chromatogram of three LPE batches revealed high similarity, and the total polyphenol content was consistently comparable, indicating the extraction method’s stability and reliability. The LPE batch with the highest polyphenol content was selected for further experimentation.

### 2.3. Determination of Total Polyphenol Content of LPE

The total polyphenol content of LPE was determined using the Folin–Ciocalteu method [28]. A standard gallic acid solution (0.1 mg/mL) was prepared. Varying volumes (0.1, 0.2, 0.3, 0.4, 0.5, 0.7 mL) of this solution were transferred into 10 mL test tubes, to which 0.5 mL of Folin–Ciocalteu reagent and 1.5 mL of 15% Na2CO3 solution were added. Then, the mixture was brought to a volume of 10 mL with distilled water, mixed thoroughly, and incubated at 75 °C for 15 min. The absorbance was recorded at 760 nm. A standard curve was constructed using gallic acid concentration on the *x*-axis and absorbance on the *y*-axis. The LPE sample solution (0.1 mg/mL) was prepared and analyzed in a similar manner to the gallic acid standards. Total polyphenol content was calculated using the linear regression equation from the standard curve, and expressed in milligrams of gallic acid equivalents per gram of LPE (mg GAE/g LPE). This procedure was conducted three times to determine an average value for the total polyphenol content in LPE.

### 2.4. Determination of (−)-Epicatechin Content of LPE

The (−)-epicatechin content in LPE was quantified using a high-performance liquid chromatograph (HPLC) (Agilent, Palo Alto, CA, USA). Samples of LPE and (−)-epicatechin were precisely measured, dissolved in methanol, sonicated for complete dissolution, and then filtered through a micropore filter membrane. The chromatographic conditions were as follows: a ZORBAX SB-C18 column at 30 °C, a flow rate of 0.7 mL/min, an injection volume of 10 μL, mobile phase A (acetonitrile), mobile phase B (0.4% phosphoric acid solution), and a gradient elution process. Each sample was analyzed three times, with the average value indicating the (−)-epicatechin content in the extract.

### 2.5. Animal Experiment Design

The mice were housed at the Laboratory Animal Center of the Guangxi Institute of Botany (Guilin, China), in conditions maintaining a 12-h light/dark cycle, a temperature ranging from 22 to 24 °C, and a humidity between 55 and 70%. Unrestricted access to water and a standard diet were provided. The experimental protocol adhered to international standards for the use and welfare of laboratory animals, and received approval from the research ethics committee of the Guangxi Institute of Botany, Guangxi Zhuang Autonomous Region, and the Chinese Academy of Science (GXZW-2022122001). After a 7-day acclimation period, 12 wild-type db/dm mice were allocated to the blank control group (BC), and 36 db/db diabetic mice were equally divided into three groups based on fasting blood glucose (FBG) levels: the model control group (MC), the low-dose LPE group (L-LPE), and the high-dose LPE group (H-LPE), with 12 mice in each group. LPE was prepared in distilled water and administered orally via gavage at doses of 200 mg/kg (L-LPE) or 400 mg/kg (H-LPE), or an equivalent volume of distilled water for the BC and MC, daily for 8 weeks. The mice were monitored weekly for weight changes, with doses adjusted accordingly.

### 2.6. Determination of FBG and Glucose Tolerance in Mice

FBG levels were monitored weekly. Following an 8-h fast, blood glucose levels were assessed using a model 580 blood glucose meter (Yuwell, Suzhou, China) and its corresponding test strips. In the 8th week, FBG levels were again measured in all groups using the same methodology. Additionally, glucose tolerance was evaluated, with glucose measurements taken at 0.5, 1, and 2 h after administering 2.5 g/kg of glucose intraperitoneally.

### 2.7. Animal Handling

At the conclusion of the 56-day treatment period, the mice were anesthetized with sodium pentobarbital. Upon reaching unconsciousness, they were euthanized through cervical dislocation, and subsequently, their livers and blood were collected. The weight of each liver was meticulously recorded, and the liver index was calculated as follows: Liver index = liver weight (mg)/mouse weight (g). The blood was placed in sterilized EP tubes and centrifuged at 3500 rpm for 10 min at 4 °C to obtain the serum, which was then preserved at −20 °C. The same part of the liver was homogenized using a glass homogenizer, and the homogenate was centrifuged at 3500× *g* for 10 min at 4 °C. The supernatant was stored at −80 °C, while the remainder of the liver tissue was kept in liquid nitrogen for Western blot analysis.

### 2.8. Determination of Biochemical Indexes

The levels of alanine transaminase (ALT) and aspartate transaminase (AST) in the blood were measured using the specified kits (Jiancheng, Nanjing, China). The activities of SOD and the content of MDA in both the liver and blood were determined with kits from the same supplier (Jiancheng, Nanjing, China). Blood insulin concentrations were assessed following the guidelines of the kits provided by the manufacturer (Mlbio, Shanghai, China). Furthermore, levels of TNF-α, IL-6, and IL-1β in both liver and blood were quantified using kits from the manufacturer (Mlbio, Shanghai, China).

### 2.9. Extraction of Total Protein and Nucleoproteins

Total liver protein was isolated by homogenizing 50 mg of frozen liver tissue in 200 μL of RIPA lysis buffer utilizing an ultrasonic homogenizer. Following homogenization, the mixture was centrifuged at 12,000× *g* for 15 min at 4 °C, and the supernatant was collected as the total protein. The extraction of liver tissue nucleoprotein followed the protocol provided by the kit (Beyotime, Shanghai, China).

### 2.10. Western Blot Analysis

A sample of the protein supernatant was assessed for protein concentration using the kit (Beyotime, Shanghai, China). The remainder of the supernatant was combined with loading buffer and subjected to boiling water for 5 min to denature the proteins. Then, SDS-PAGE gel electrophoresis was conducted on samples each containing 30 μg of protein, tailored to the molecular weight of the target proteins. For the detection of protein expression, enhanced chemiluminescence was employed. Protein band intensities were semi-quantitatively analyzed using an image processing system, with grayscale values indicating protein expression levels.

### 2.11. Statistical Analysis

All experimental data are presented in terms of mean ± standard deviation. Statistical analyses were performed using GraphPad Prism 8 (GraphPad, San Diego, CA, USA). The regression analysis used simple residuals, which were adjusted by the predicted values, with standardized residuals against the observed values to detect outliers. Both outliers and missing data were excluded from the analysis. The normality of the data distribution was assessed using the Kolmogorov–Smirnov test, while the homogeneity of variances was evaluated with Bartlett’s test. For comparisons across multiple groups, one-way ANOVA was utilized, with subsequent post hoc comparisons carried out using Tukey’s test. A significance level of *p* < 0.05 was used to determine statistical significance.

## 3. Results and Discussion

### 3.1. Content of Total Polyphenols and (−)-Epicatechin in LPE

Table 1 indicates that LPE contains 68.37% total polyphenols and 12.92% (−)-epicatechin. Considering the hypoglycemic properties of polyphenols and reports in the literature of (−)-epicatechin improving glucose consumption in HepG2 cells [24], LPE holds promise for the treatment of type 2 diabetes.

### 3.2. Effect of LPE on Body Weight and 24 h Food Intake in Mice

**Figure 1:**
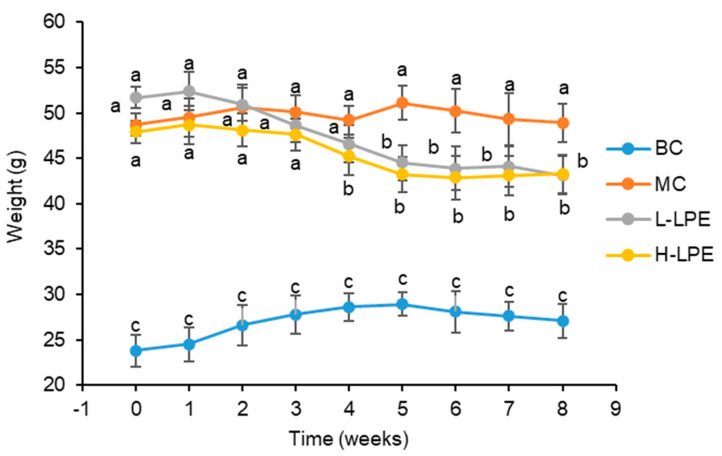
Effects of LPE on body weight in mice. Values are expressed as mean ± standard deviation (*n* = 12 in each group). The values with different letters (a, b, or c) are significantly different (*p* < 0.05) between each group.

Changes in body weight serve as a crucial indicator of the physiological impact of pharmacological interventions [29]. A variety of factors, such as drug toxicity or the inherent weight loss properties of the compound, can influence weight gain. Therefore, monitoring weight changes is essential. As illustrated in Figure 1, the weights of the BC and MC mice remained stable throughout the study. Conversely, db/db mice treated with both low-dose and high-dose LPE experienced a gradual decrease in weight over the course of the experiment. While the MC mice showed a significant weight increase compared to the BC mice (*p* < 0.05), the weight of mice in the L-LPE did not significantly differ from the MC mice in the first 4 weeks (*p* > 0.05); however, from the fifth week onward, a significant decrease was noted (*p* < 0.05). Similarly, weight loss in the H-LPE mice was not significantly different from the MC mice in the initial 3 weeks (*p* > 0.05), but showed a significant reduction in the following 5 weeks (*p* < 0.05). Nevertheless, a dose–response relationship was not established between the low and high doses of LPE for weight reduction. Previous studies have confirmed LPE’s non-toxicity in mice at doses up to 400 mg/kg [30], indicating that the weight loss may be due to LPE’s slimming effects. Table 2 shows that the 24-h food intake of MC mice was significantly higher than that of BC mice (*p* < 0.05). Compared to the MC mice, 24-h food intake in the L-LPE and H-LPE mice resulted in a minor decrease (*p* > 0.05), implying that the weight loss induced by LPE is not related to reduced food intake.

### 3.3. Effect of LPE on FBG in Mice

**Figure 2:**
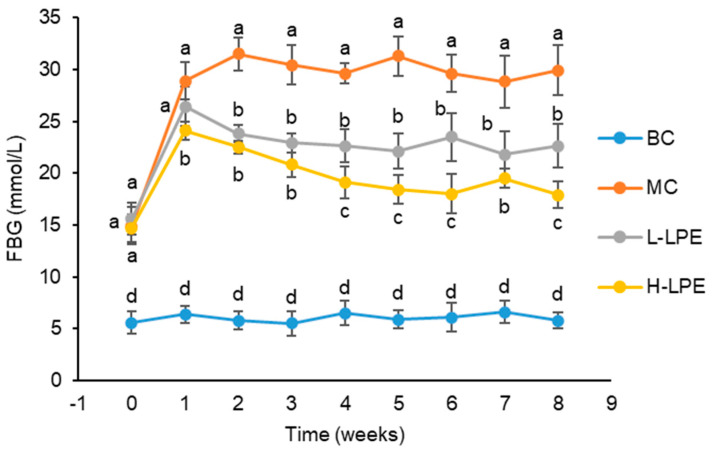
Effects of LPE on FBG in mice. Values are expressed as mean ± standard deviation (*n* = 12 in each group). The values with different letters (a, b, c, or d) are significantly different (*p* < 0.05) between each group.

Figure 2 shows that the FBG levels of db/dm mice in the BC remained stable throughout the experiment. In contrast, the FBG levels in db/db mice in the MC were significantly higher than those in the BC from the beginning of the study (7 weeks old), maintaining elevated levels from 1 week into the experiment (8 weeks old) until its conclusion (15 weeks old). In the L-LPE, the db/db mice experienced a reduction in FBG levels after one week of treatment compared to the MC. Although this reduction was not statistically significant (*p* > 0.05), it demonstrated a significant decrease from the second week of treatment until the end of the eighth week (*p* < 0.05). The db/db mice in the H-LPE had statistically significant lower FBG levels than the MC from the first week of treatment until the end of the experiment (*p* < 0.05). As shown in Figure 2, H-LPE was more efficacious in reducing FBG levels than L-LPE, showing a significant dose–response relationship.

### 3.4. Effect of LPE on Glucose Tolerance in Mice

**Figure 3:**
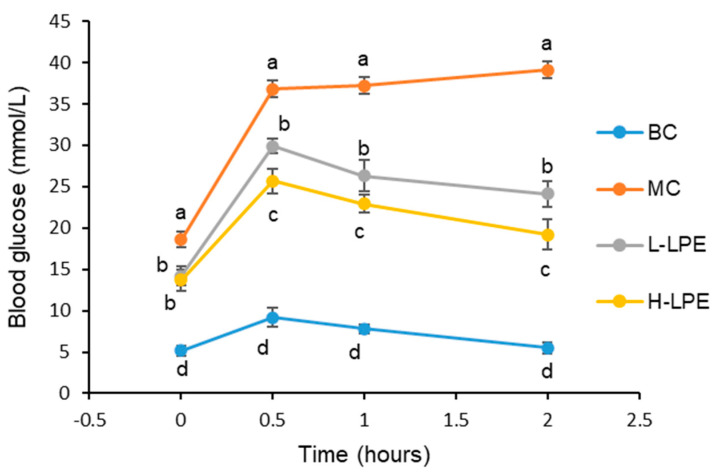
Effects of LPE on glucose tolerance in mice. Values are expressed as mean ± standard deviation (*n* = 12 in each group). The values with different letters (a, b, c, or d) are significantly different (*p* < 0.05) between each group.

Figure 3 depicts the glucose tolerance test outcomes for the BC, where db/dm mice exhibited a moderate increase in blood glucose levels after receiving an intraperitoneal injection of 2.5 g/kg glucose. The blood glucose levels peaked at 0.5 h, then declined gradually, returning to baseline after 2 h. In contrast, db/db mice in the MC showed a swift rise in blood glucose levels immediately after the injection, reaching near-peak levels at 0.5 h, and continued to increase slightly at 1 and 2 h. db/db mice in both the H-LPE and L-LPE groups experienced a sharp increase in blood glucose levels following the 2.5 g/kg glucose injection, with a peak at 0.5 h. Compared to the MC, these groups exhibited a significant reduction in blood glucose levels, achieving an earlier peak. By 1 h, blood glucose levels in the L-LPE mice had decreased by 29.3% and in the H-LPE mice by 38.4%, relative to the MC mice. At 2 h, the reductions were 38.4% in the L-LPE and 50.9% in the H-LPE. The glucose tolerance test indicated dose-dependence with LPE treatment in the db/db mice, highlighting LPE’s potential to enhance glucose tolerance in these mice.

### 3.5. Effect of LPE on Serum Insulin in Mice

**Figure 4:**
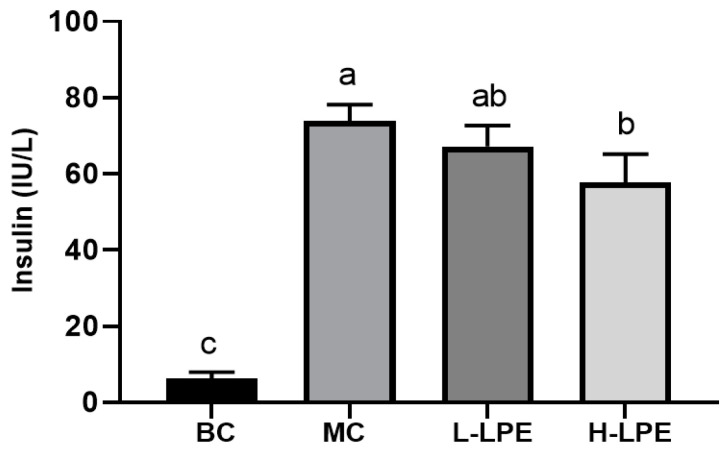
Effects of LPE on serum insulin levels in mice. Values are expressed as mean ± standard deviation (*n* = 12 in each group). The values with different letters (a, b, or c) are significantly different (*p* < 0.05) between each group.

Insulin resistance, characterized by diminished insulin sensitivity in peripheral cells, is a pathological state. db/db mice, which serve as a model for spontaneous obesity and diabetes due to a deficiency in the leptin receptor gene, exhibit significantly elevated insulin levels, as confirmed by prior studies [31]. Figure 4 illustrates that the insulin levels in MC mice were higher than those in BC mice, consistent with existing research [31], and indicative of pronounced insulin resistance in the db/db mice. In the L-LPE mice, there was a slight decrease in insulin levels compared to the MC mice, though this reduction was not statistically significant (*p* > 0.05). Conversely, the H-LPE mice demonstrated a significant decrease in insulin levels (*p* < 0.05). Abnormal glucose tolerance is directly linked to either inadequate insulin production post-meal or reduced insulin sensitivity. As noted in Section 3.4, LPE enhanced glucose tolerance in the db/db mice. The study also revealed that the abnormally high insulin levels in db/db mice were alleviated by LPE treatment, indicating LPE’s potential in restoring insulin sensitivity in peripheral cells and diminishing insulin resistance.

### 3.6. Effects of LPE on Liver Function in Mice

**Figure 5:**
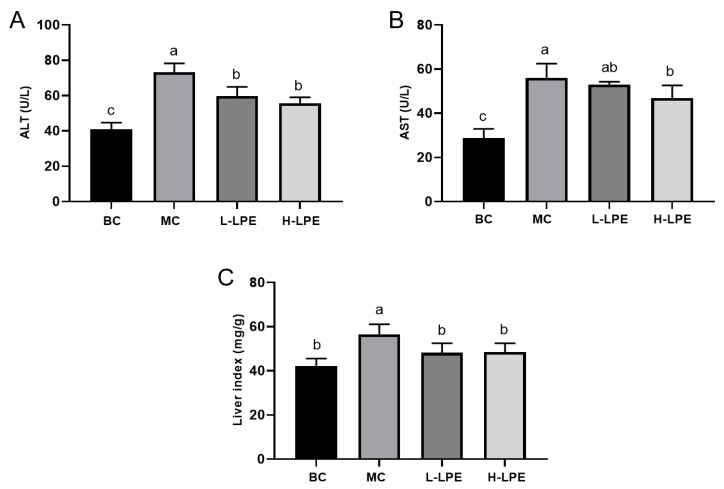
Effects of LPE on liver function in mice. (**A**) ALT activity. (**B**) AST activity. (**C**) Liver index. Values are expressed as mean ± standard deviation (*n* = 12 in each group). The values with different letters (a, b, or c) are significantly different (*p* < 0.05) between each group.

The liver index has been widely adopted as an index to assess the health status of the liver [32]. ALT and AST are widely employed in liver function tests, which are released into the bloodstream as hepatocytes are damaged, making them valuable markers for assessing liver function [33]. Figure 5 reveals that serum ALT and AST levels, as well as the liver index in MC db/db mice, were significantly higher than those in the BC db/dm mice, indicating liver damage in the db/db mice. In comparison to MC mice, the liver index in the L-LPE showed a reduction, with a decrease in ALT levels (*p* < 0.05) but no significant alteration in AST levels (*p* > 0.05). In the H-LPE, the liver index was further reduced, and both ALT and AST levels significantly decreased (*p* < 0.05), suggesting that LPE may offer liver protection in db/db mice, and merits further research.

### 3.7. Effect of LPE on Inflammatory Factors in Mice

**Figure 6:**
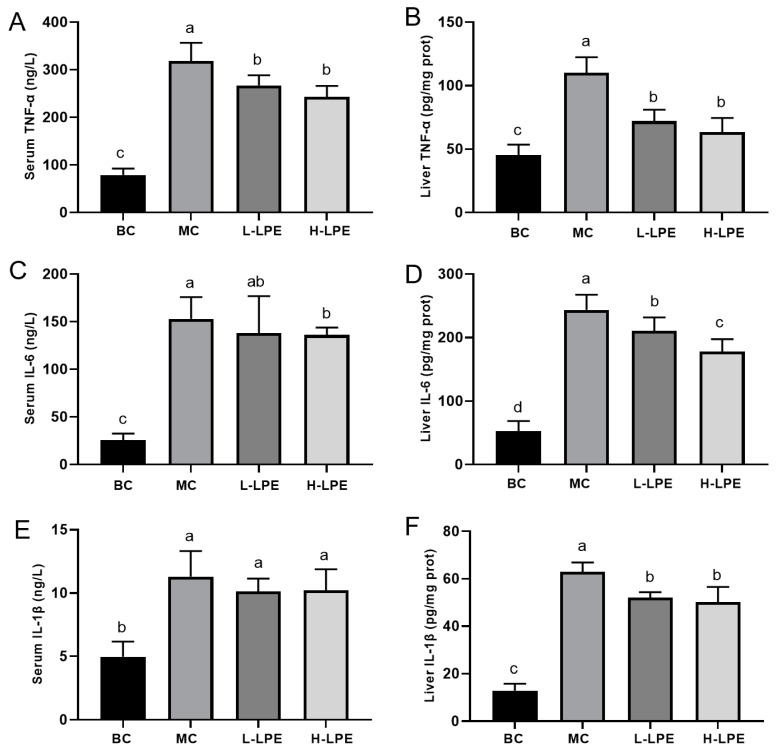
Effects of LPE on inflammatory cytokines in mice. (**A**) Serum TNF-α content. (**B**) Liver TNF-α content. (**C**) Serum IL-6 content. (**D**) Liver IL-6 content. (**E**) Serum IL-1β content. (**F**) Liver IL-1β content. Values are expressed as mean ± standard deviation (*n*= 12 in each group). The values with different letters (a, b, c, or d) are significantly different (*p* < 0.05) between each group.

Type 2 diabetes mellitus, a metabolic disease, is often accompanied by inflammation [16]. Elevated levels of inflammatory factors are commonly observed in patients with type 2 diabetes [34,35]. Figure 6 reveals increased levels of TNF-α, IL-6, and IL-1β in both the serum and liver of mice in the MC compared to the BC mice, indicating an inflammatory state in db/db mice, which supports earlier research [36]. In the L-LPE mice, TNF-α levels in both the serum and liver of mice were significantly reduced (*p* < 0.05) in the MC mice. A slight decrease in IL-6 and IL-1β levels in the serum of L-LPE mice was observed (*p* > 0.05), but the reductions in IL-6 and IL-1β levels in the liver were statistically significant (*p* < 0.05) compared to the MC mice. In the H-LPE, both serum and liver levels of TNF-α and IL-6 in mice were significantly lower compared to the MC mice (*p* < 0.05); meanwhile, the decrease in blood levels of IL-1β was not significant (*p* > 0.05), and the liver levels of IL-1β were significantly reduced (*p* < 0.05). These results suggest that LPE can reduce the inflammatory response in db/db mice.

### 3.8. Effect of LPE on Antioxidant Capacity of Mice

**Figure 7:**
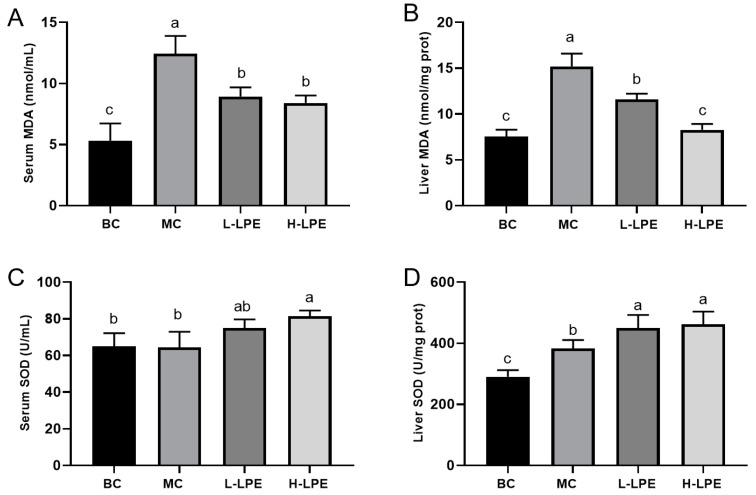
Effect of LPE on antioxidant capacity of mice. (**A**) Serum MDA content. (**B**) Liver MDA content. (**C**) Serum SOD activity. (**D**) Liver SOD activity. Values are expressed as mean ± standard deviation (*n* = 12 in each group). The values with different letters (a, b, or c) are significantly different (*p* < 0.05) between each group.

Oxidative stress is intrinsically linked to elevated blood glucose levels [10]. Previous studies have documented the potent antioxidant properties of LPE [19,26]. This study measured the SOD activity and MDA content associated with oxidative stress in the blood and liver of mice. SOD primarily functions to convert superoxide anions (O_2_^•−^) to oxygen (O_2_) and hydrogen peroxide (H_2_O_2_), preventing their accumulation and cellular oxidative damage [37]. MDA, a byproduct of lipid peroxidation, serves as an indicator of oxidative stress levels [38]. Figure 7A,B indicate that MDA levels in the blood and liver of db/db mice in the MC were higher compared to db/dm mice in the BC. In contrast, LPE treatment resulted in reduced MDA levels, suggesting that LPE can counteract oxidative stress induced by prolonged hyperglycemia. The body’s response to oxidative stress includes the upregulation of antioxidant enzymes like SOD, enhancing antioxidant defenses and protecting cells from oxidative harm [39], which was confirmed by our experimental results. As depicted in Figure 7C,D, liver SOD activity in db/db mice of the MC was significantly higher than in db/dm mice of the BC (*p* < 0.05). In the LPE-treated groups, SOD activity in both the liver and blood of db/db mice was further elevated compared to the MC mice, indicating that LPE enhances the activity of antioxidant enzymes, bolstering the body’s capacity to resist oxidative damage. This suggests that while the body’s innate protective mechanisms against oxidative stress are valuable, they may be insufficient to fully counteract external oxidative insults, and LPE’s role in upregulating antioxidant enzyme activity further mitigates oxidative stress.

### 3.9. Effect of LPE on AMPK Pathway

**Figure 8:**
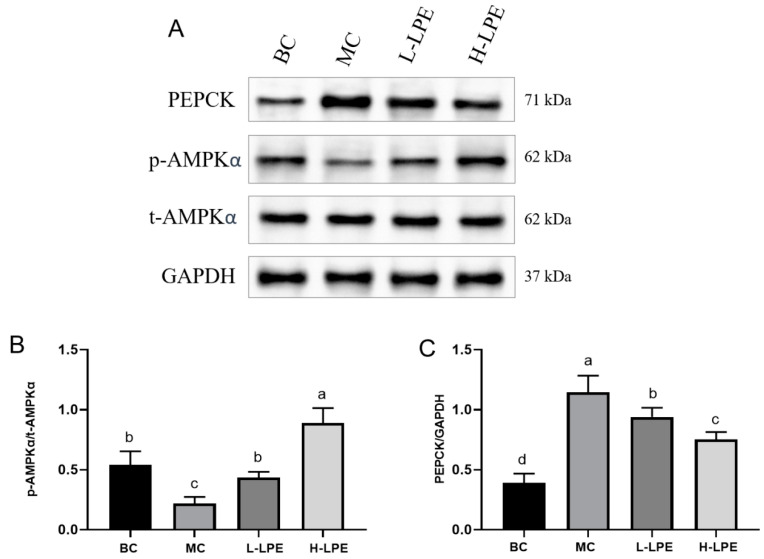
Effects of LPE on the protein expression of AMPK signaling pathway in mice. (**A**) The protein expressions of t-AMPKα, p-AMPKα, and PEPCK. (**B**) Quantification of protein levels of p-AMPKα/t-AMPKα. (**C**) Quantification of protein levels of PEPCK. Values are expressed as mean ± standard deviation (*n* = 3 in each group). The values with different letters (a, b, c, or d) are significantly different (*p* < 0.05) between each group.

The liver regulates glucose homeostasis through a variety of glucose metabolism-related signaling pathways [40]. AMPK is now widely recognized as the crucial protein responsible for balancing energy, and activated AMPK regulates a variety of metabolic processes, including enhanced glycolysis and reduced gluconeogenesis [41]. Glucogenesis is the process of converting a non-carbohydrate substrate into glucose or glycogen. The most important organ for gluconeogenesis is the liver, where PEPCK is the key rate-limiting enzyme in the process, and its expression determines the rate of gluconeogenesis [42,43]. Thus, managing gluconeogenesis is vital for treating type 2 diabetes. Evidence suggests that AMPK expression is diminished in tissues like the liver, adipose, and muscle in individuals with type 2 diabetes and in insulin-resistant animal models [44]. As shown in Figure 8, studies have shown a reduced p-AMPKα/t-AMPKα ratio in db/db mice liver, which is confirmed by our results [45]. In our study, db/db mice had a decreased p-AMPKα/t-AMPKα ratio in the liver compared to their wild-type db/dm mice. Further examination of PEPCK, a downstream protein of the AMPK pathway, revealed an abnormal elevation in db/db mice, pointing to a significant gluconeogenesis imbalance, potentially contributing to heightened blood glucose levels. LPE intervention upregulated the p-AMPKα/t-AMPKα ratio in db/db mice, indicating reactivation of suppressed AMPK. In addition, PEPCK protein expression was reduced, suggesting that LPE-induced AMPK activation inhibited PEPCK expression, and thus moderated the gluconeogenesis response. These results imply that LPE functions as an AMPK agonist, curtailing liver gluconeogenesis through AMPK activation in db/db mice, thereby lowering blood glucose levels. 

### 3.10. Effect of LPE on Nrf2 Signaling Pathway

**Figure 9:**
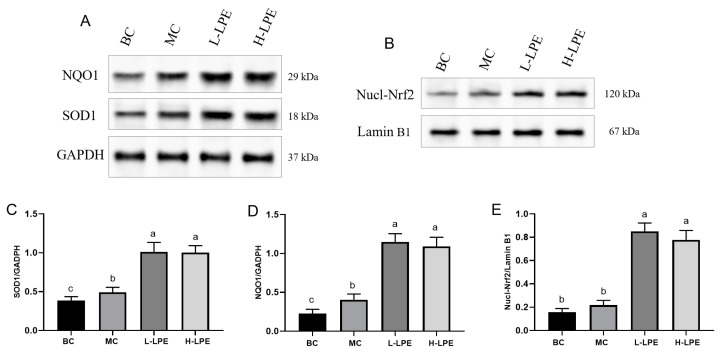
Effects of LPE on the protein expression of Nrf2 signaling pathway in mice. (**A**) The protein expressions of SOD1 and NQO1. (**B**) The protein expression of Nucl-Nrf2. (**C**) Quantification of protein levels of SOD1. (**D**) Quantification of protein levels of NQO1. (**E**) Quantification of protein levels of Nucl-Nrf2. Values are expressed as mean ± standard deviation (*n* = 3 in each group). The values with different letters (a, b, or c) are significantly different (*p* < 0.05) between each group.

The Nrf2 signaling pathway is instrumental in managing oxidative stress [13], with Nrf2 primarily residing in the cytoplasm under conditions of oxidative equilibrium [46,47]. Oxidative stress triggers Nrf2 translocation from the cytoplasm to the nucleus, binding to the antioxidant response element (ARE) and initiating transcription of downstream antioxidants like SOD, HO-1, and NQO1 [48,49]. Figure 9B,E reveal augmented nuclear Nrf2 protein expression in the MC mice compared to the BC mice, indicating an enhanced oxidative stress response. The administration of LPE led to a further increase in nuclear Nrf2 expression, suggesting LPE’s efficacy in facilitating Nrf2’s nuclear migration. NQO1 is critical for cellular defense, facilitating electron transfer between quinones and NAD(P)H to mitigate oxidative stress impacts [50], alongside SOD, a crucial antioxidant enzyme. Figure 9A,C,D indicate increased NQO1 and SOD1 protein expressions in the MC mice compared to the BC mice, with further elevation in the LPE-treated groups. This implies that hyperglycemia-induced oxidative stress promotes Nrf2 nuclear entry, initiating antioxidant factor expression, and LPE further boosts antioxidant factor expression, enhancing the body’s antioxidant capacity and mitigating hyperglycemia-induced damage. The results of Section 3.8 align with these results, showing LPE increased SOD activity and reduced MDA content in db/db mice, alleviating oxidative stress. Additionally, the activation of AMPK is purported to activate Nrf2, thereby augmenting the transcription of intracellular antioxidant genes [51], as demonstrated in Section 3.9, where LPE is shown to act as an AMPK agonist, possibly elucidating LPE’s mechanism in promoting Nrf2’s entry into the nucleus. 

### 3.11. Effect of LPE on NF-κB

**Figure 10:**
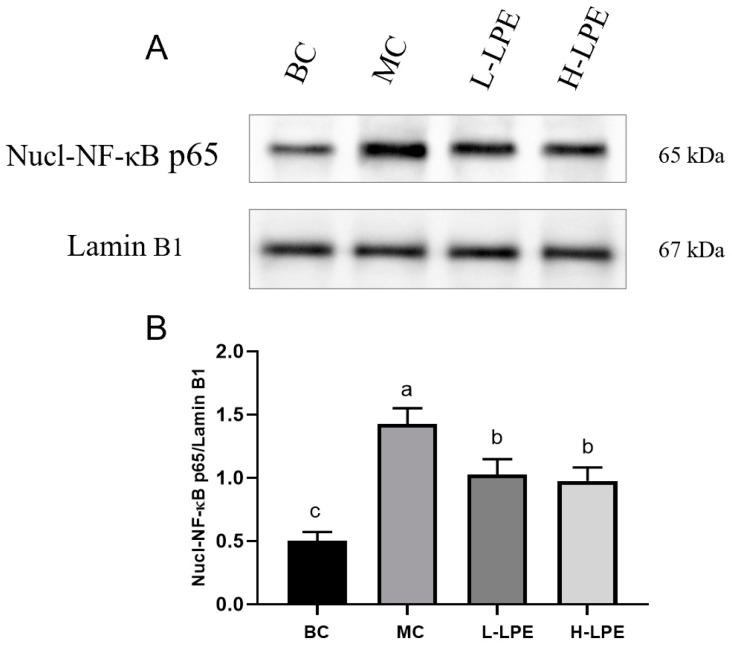
Effects of LPE on the protein expression of NF-κB in mice. (**A**) The protein expression of Nucl-NF-κB p65. (**B**) Quantification of protein levels of Nucl-NF-κB p65. Values are expressed as mean ± standard deviation (*n* = 3 in each group). The values with different letters (a, b, or c) are significantly different (*p* < 0.05) between each group.

NF-κB is a critical inflammatory signaling pathway, which triggers inflammation upon activation [18]. Under conditions of homeostasis, NF-κB bound to inhibitory proteins; however, upstream signals such as TNF-α and IL-6 can prompt its nuclear entry, initiating an inflammatory response. Figure 10A,B illustrate that nuclear NF-κB protein expression in db/db mice of the MC was higher compared to db/dm mice in the BC, indicating that hyperglycemia-induced inflammation activates NF-κB. In the LPE-treated groups, nuclear NF-κB expression was reduced, suggesting LPE’s inhibitory effect on NF-κB activation. Section 3.7 aligns with these findings, showing that LPE reduces TNF-α, IL-6, and IL-1β levels in db/db mice, which may be attributable to its suppression of NF-κB nuclear entry. Research has demonstrated that activated AMPK can inhibit the NF-κB pathway via silent information regulator 1 (SIRT1) activation [52,53]. Our prior studies confirmed LPE as an AMPK agonist, suggesting it may inhibit NF-κB through AMPK pathway activation.

## 4. Conclusions

In this study, a polyphenol-based extract from lychee peels was obtained, with (−)-epicatechin identified as its primary component. Db/db mice, characterized by disrupted glucose metabolism, insulin resistance, chronic inflammation, and oxidative stress, showed improvement in these conditions upon treatment with LPE. Mechanistic investigations linked the efficacy of LPE in managing type 2 diabetes to the activation of the AMPK pathway and modulation of the Nrf2 and NF-κB signaling pathways. Activation of AMPK by LPE led to the inhibition of PEPCK protein expression, reducing blood glucose levels by suppressing hepatic gluconeogenesis. Additionally, LPE mitigated oxidative stress through AMPK activation, which facilitated Nrf2’s nuclear translocation and initiated the transcription of the downstream antioxidants SOD and NQO1. LPE also inhibits inflammatory responses by reducing NF-κB nuclear entry through AMPK activation. Although LPE has shown positive effects in improving type 2 diabetes, the challenge of generalizing results from animal models to human patients remains a critical issue. Direct translation of observations in mice to humans frequently disappoints, for reasons including discrepancies in complexity and regulation between species. Differences in the lifestyles of humans and mice may also impact the outcomes of transformation. The etiology of human type 2 diabetes is multifaceted, encompassing dietary, lifestyle, and genetic factors. The db/db mice, characterized by a defect in the leptin receptor gene, serve as an animal model for studying this complex disease. However, it is important to note that direct translation of findings from studies involving db/db mice to humans may not be feasible. Repetition of experimental findings in different type 2 diabetes models may help to ensure that the observed effect is generalizable to a broader context. Systems biology and machine learning can be used to translate relationships across species. Instead of attempting to “humanize” animal experimental models, greater success may be obtained by humanizing computational models derived from animal experiments. This approach offers a promising solution to the current predicament. Physical activity has been demonstrated to be effective in the prevention of type 2 diabetes and in promoting weight reduction. However, this study did not investigate the impact of LPE on physical activity in mice. Therefore, it is recommended that future research should focus on conducting relevant studies in this area. LPE did not appear to have an impact on the food intake of mice. However, it is still unknown whether it reduces the digestible energy in mice. Therefore, it is necessary to conduct tests on the fecal output and energy content in the feces of mice, in order to determine the effect of LPE on digestible energy in mice. The polyphenols have been demonstrated to enhance thermogenesis, suggesting that LPE probably attenuates obesity in mice by increasing thermogenesis through the activation of brown adipose tissue. This warrants further investigation. This research enhances the application and development prospects of lychee peel.

## Figures and Tables

**Table 1:** Bioactive components of LPE.

Item	LPE
Total polyphenols (%)	68.37 ± 1.98
(−)-epicatechin (%)	12.92 ± 1.12

Values are presented as the mean ± standard deviation of triplicate experiments.

**Table 2:** Effect of LPE on 24 h food intake of mice.

Group	24 h Food Intake (g)
BC	3.25 ± 0.42 ^b^
MC	6.38 ± 0.73 ^a^
L-LPE	6.21 ± 0.68 ^a^
H-LPE	6.10 ± 0.81 ^a^

Values are expressed as mean ± standard deviation (*n* = 12 in each group). Differences between groups, indicated by distinct letters (a or b), were considered statistically significant (*p* < 0.05).

## Data Availability

The original contributions presented in the study are included in the article, further inquiries can be directed to the corresponding authors.
